# Redox-governed charge doping dictated by interfacial diffusion in two-dimensional materials

**DOI:** 10.1038/s41467-019-12819-w

**Published:** 2019-10-30

**Authors:** Kwanghee Park, Haneul Kang, Seonghyun Koo, DaeEung Lee, Sunmin Ryu

**Affiliations:** 1 Department of Chemistry, Pohang University of Science and Technology (POSTECH), Pohang, Gyeongbuk 37673 Korea; 2 Department of Applied Chemistry, Kyung Hee University, Yongin, Gyeonggi 17104 Korea; 3 Division of Advanced Materials Science, Pohang University of Science and Technology (POSTECH), Pohang, Gyeongbuk 37673 Korea

**Keywords:** Optical properties and devices, Electron transfer, Raman spectroscopy, Two-dimensional materials

## Abstract

Controlling extra charge carriers is pivotal in manipulating electronic, optical, and magnetic properties of various two-dimensional materials. Nonetheless, the ubiquitous hole doping of two-dimensional materials in the air and acids has been controversial in its mechanistic details. Here we show their common origin is an electrochemical reaction driven by redox couples of oxygen and water molecules. Using real-time photoluminescence imaging of WS_2_ and Raman spectroscopy of graphene, we capture molecular diffusion through the two-dimensional nanoscopic space between two-dimensional materials and hydrophilic substrates, and show that the latter accommodate water molecules also serving as a hydrating solvent. We also demonstrate that HCl-induced doping is governed by dissolved O_2_ and pH in accordance with the Nernst equation. The nanoscopic electrochemistry anatomized in this work sets an ambient limit to material properties, which is universal to not only 2D but also other forms of materials.

## Abstract (web summary)

Manipulation of charge carriers is promising for tuning electronic, optical and magnetic properties in two-dimensional materials, but mechanistic details are not fully understood. Here, the authors report that ambient redox reactions govern charge transfer doping in graphene and tungsten disulfide.

## Introduction

Reduction in dimensions has seen many scientific discoveries in various metallic and semiconducting low-dimensional materials during the past four decades. Because of their high fraction of surface atoms, in particular, various material properties of two-dimensional (2D) materials are greatly affected by charge exchange with neighboring chemical entities or environments. When exposed to alkali metals^1^ or halogens^2^, for example, the Fermi level (E_F_) of graphene swings by several tenths of 1 eV with a substantial change in electrical conductivity^3^ or optical absorption^4^. Such chemical modification of the electronic structure allows detection of even a single molecule that adsorbs on graphene in a transistor form^5^. The charge exchanges can be described by a simple donor–acceptor model by assuming a significant difference in their electron affinities. Despite the apparent simplicity, however, there is a clear lack of understanding in the ubiquitous chemical interactions of 2D materials. The first graphene transistor was reported to be doped with hole carriers originating from unknown chemical entities^6^. Optical confirmation soon followed this observation, but with no complete mechanistic understanding^7^. While various acids were exploited to inject hole carriers in graphene, their exchange mechanism is not clear^8^. All of these underline that the spontaneous charge transfer between 2D materials and the environment is far from being understood, which hampers both fundamental research and application of 2D materials. In this work, we elucidate that the charge exchange is driven by redox couples of oxygen and water molecules, originally proposed for surface conduction of diamonds^9,10^, and that the redox reaction is confined within the nanoscopic space between 2D materials and hydrophilic substrates. By exploiting environment-controlled in situ spectromicroscopy, we captured molecular diffusion through the interstitial 2D space and revealed that photoluminescence (PL) of WS_2_ and lattice vibration of graphene, both as an indicator of charge density, are dictated by oxygen and water contents and their spatiotemporal distribution.

## Results

### Chemically modulated ionization of excitons

**Fig. 1:**
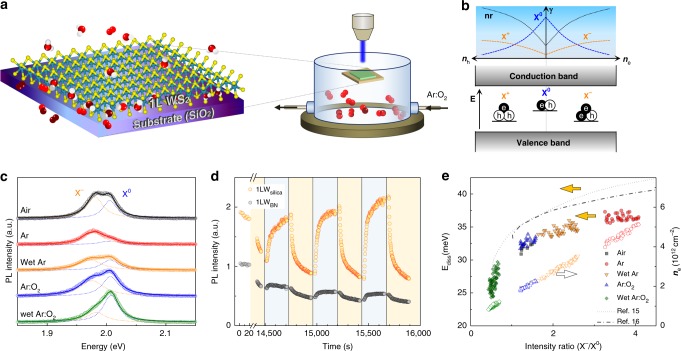
Chemically modulated ionization of excitons. **a** In situ optical measurements of single-layer (1L) WS_2_ supported on SiO_2_ (1LW_silica_) located in a controlled gaseous environment. The scheme illustrates 1LW_silica_ interacting with O_2_ and water in a redox reaction. **b** Electronic energy bands of WS_2_ with neutral (X^0^) and charged (X^+^ and X^−^) excitonic states (bottom panel); schematic representation of their radiative and nonradiative (nr) decay rates (*γ*) given as a function of charge density (***n***_e_ and ***n***_h_) (ref. ^14^) (upper panel). **c** Photoluminescence (PL) spectra of WS_2_ in various gas environments. Solid lines are double Lorentzian fits representing X^0^ and X^−^. Relative humidity for wet gases was 45%. **d** Time-lapse measurements of PL intensity of 1LW_silica_ and 1LW_BN_ in response to changes in gas environments: ambient air (white box), Ar (yellow box), and Ar:O_2_ = 4:1 (blue box). **e** Equivalence between chemical (filled symbols) and electrical (lines, refs. ^15,16^) modulations of charge density in WS_2_, where trion dissociation energy (*E*_diss_) in the left ordinate was given as a function of PL intensity ratio (X^−^/X^0^). The electron density (***n***_e_) in the right ordinate (open symbols) was determined for the filled symbols according to the mass action law for excitons and trions (Supplementary Note 1)

Single-layer WS_2_ (1LW_silica_) was mechanically exfoliated onto SiO_2_/Si substrates (see the “Methods” section) and served as a model 2D system with precise control over gas environments (Fig. 1a and Supplementary Fig. 1). Photogenerated excitons (X^0^) have substantial binding energy due to dimensionally restricted screening^11^ and interact with excess charge carriers to form charged excitons or trions (X^**±**^) with additional stabilization as depicted in Fig. 1b^12^. As shown in Fig. 1c, the PL spectra of 1LW_silica_ in the ambient air typically showed two peaks, each originating from excitons (X^0^ at *E*_X0_ = 2.01 eV) and negative trions (X^−^ at E_X−_ = 1.98 eV), the latter of which derived from defect-related native charge carriers^12^. By assuming that the ionization reactions of excitons into trions are governed by the law of mass action^13^, their PL intensity ratio (*I*_X−_/*I*_X0_) serves as a quantitative measure for the density of charge carriers that are modulated by chemical interaction between WS_2_ and the environments (Supplementary Note 1). When the optical gas cell was purged with an Ar gas, *I*_X−_/*I*_X0_ increased noticeably, which indicated an increase in electron density (***n***_e_). The concomitant decrease in the total intensity (*I*_t_ = *I*_X−_ + *I*_X0_) is due to the increasing importance of the nonradiative decay channel at higher ***n***_e_^14^. Water vapor in the Ar gas, however, reduced ***n***_e_ when judged from a noticeable decrease in *I*_X−_/*I*_X0_. Exposure to dry or humid O_2_ led to an even more considerable decrease in the ratio, and thus further depletion of negative carriers. Time-lapse measurements in Fig. 1d showed that the total PL intensity varied reversibly between Ar and O_2_ atmospheres, which confirmed the molecular origin of the charge modulation. The dissociation energy of the trions (*E*_diss_ = *E*_X0_ − E_X−_) showed a strong correlation with the intensity ratio over the entire atmospheric conditions (Fig. 1e). The overall trend is similar to what was observed in electrically gated measurements^15,16^ as shown in Fig. 1e. Since *E*_diss_ is linearly dependent on the Fermi level^12^, smaller *E*_diss_ for the oxygenic or humid atmosphere indicates significant depletion of electrons induced by hole doping of WS_2_ by O_2_ or H_2_O. Judging from *E*_diss_ given as a function of *I*_X−_/*I*_X0_ in Fig. 1e, the depletion was near completion, and an injection of additional hole carriers would lead to emergence of positively charged trions. A typical difference in ***n***_e_ was ~3 × 10^13^ cm^−2^ between Ar and wet Ar:O_2_ environments (see Supplementary Note 1 for estimation of charge density). Notably, spatially resolved spectroscopy showed that the O_2_-driven rise in *I*_t_ was faster at edges than inner areas that are a few micrometers off the edges (Supplementary Fig. 2). Moreover, 1LW_BN_ (dry-transferred onto a thin hexagonal BN crystal, hBN) exhibited a much smaller *I*_t_ and O_2_ sensitivity than 1LW_silica_ (Fig. 1d). All of these spectral changes indicate that the density of charge carriers is reversibly modulated by a certain charge transfer (CT) reaction that involves O_2_ and water. The site and substrate-dependent kinetics suggested that the reactions are localized at the interface between WS_2_ and substrates.

### Interface-confined nanoscopic redox reactions

**Fig. 2:**
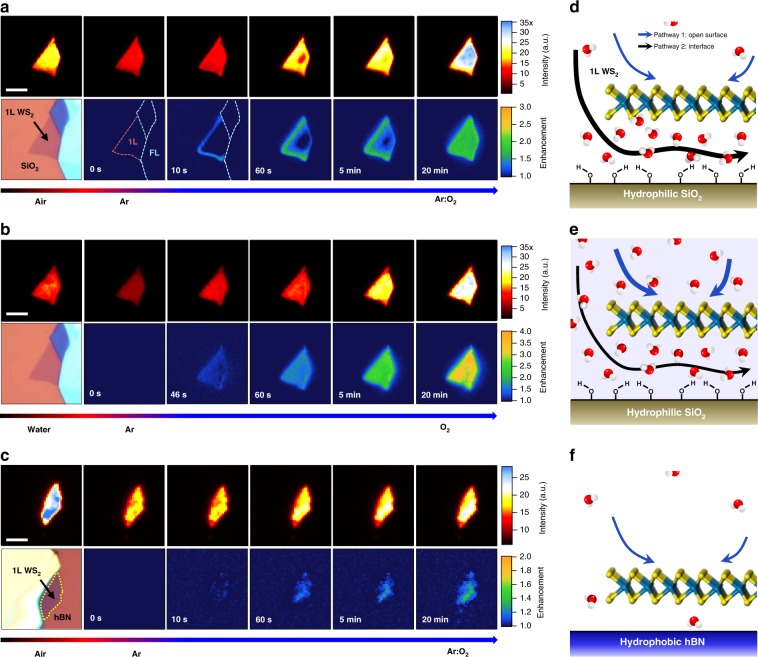
Real-time photoluminescence images of interface-confined redox reactions. **a**–**c** Wide-field PL images (top rows) and optical micrographs followed by PL enhancement images (bottom rows) of 1LW_silica_ in gas (**a**) and water (**b**), and 1LW_BN_ in gas (**c**) environments. Outlines of single and few-layer (FL) areas (dashed lines) were given in the first two enhancement images of (**a**). Exposure time was 1.5 s for each image. The enhancement images were obtained by dividing PL images with those for time zero. Samples in **a**–**c** were pre-equilibrated with Ar gas for 2 h before exposure to Ar:O_2_ mixed gas (O_2_ gas for (**b**)) at time zero. For **b**, gases were bubbled through a sparger immersed in the optical liquid cell. Scale bars: 8 (**a** and **b**) and 4 (**c**) μm. **d**–**f** Schemes for major diffusion routes of O_2_ responsible for redox reactions of 1LW_silica_ in the gas phase (**d**), 1LW_silica_ in water (**e**), and 1LW_BN_ in the gas phase (**f**). The thickness of each arrow represents the relative contribution of each pathway to the overall charge transfer reactions

Wide-field PL imaging revealed how the CT reaction evolved in space and time as shown in Fig. 2, where 1LW samples were uniformly illuminated with 514-nm laser light under controlled gas or liquid environments. In the ambient air, the intensity image of 1LW_silica_ exhibited slight local irregularities over the entire sample area of ~70 μm^2^ (Fig. 2a). The multilayer neighbors showed negligible emission due to their indirect bandgaps^17,18^. Because of the O_2_-driven CT mentioned above, the overall intensity decreased ~50% in an Ar gas and was recovered within 20 min under O_2_ flow, which is consistent with the reversibility shown in Fig. 1d. Notably, however, the recovery in Fig. 2a showed an evident edge-to-center propagation as shown in the enhancement images that are normalized by that of the deoxygenated state in Ar. Exposure for 10 s led to a sharp enhancement by ~30% at the two edges with the other two connected to the multilayers remaining silent. Within 60 s of O_2_ flow, the two edges exhibited ~100% enhancement, and the enhancement fronts moved further gradually reaching a steady state after 20 min (Supplementary Movie 1; Supplementary Fig. 3 for more examples) followed by its reversal in subsequent Ar flow (Supplementary Movie 2). In contrast, neither directional enhancement nor complete recovery was observed for 1LW_BN_ (Fig. 2c). On average, the enhancement was small (20–30%) and occurred rather uniformly, which agrees with Fig. 1d. All of these observations indicated that the CT reactants diffuse through the WS_2_–silica interface as illustrated by the thick black arrow in Fig. 2d. Noting that the upper surfaces of samples were accessible to the reactants, these results implied a crucial role of the interface or surface of silica, and led us to a mechanistic model with two essential ingredients. First, we explain that charge density of WS_2_ is modulated by the redox reaction involving oxygen and water

1 $${\rm{O}}_{2} + 4{\rm{H}}^{+} + 4{\rm{e}}^{-} \leftrightarrow 2{\rm{H}}_{2}{\rm{O}},$$

where reduction of O_2_ is accompanied by oxidation (hole doping) of WS_2_ (see Supplementary Note 2 for a detailed account on CT at the solid–liquid interface based on Gerischer model). The reaction in Eq. (1) was validated for surface transfer doping of hydrogenated diamonds^10^, the electrochemical nature of which was first proposed by Maier et al.^9^. Similar observations were also made for other material systems including GaN^19^, ZnO^19^, carbon nanotubes^20^, and graphene^21^. Second, the CT reaction is localized at the nanoscopic interface by hydrophilic surface functional groups of SiO_2_ that accommodate water molecules because of the energy gain by hydration of ionic species.

Under this model, the directional propagation of the PL enhancement (Fig. 2a and Supplementary Movies 1 and 2) indicates that the rate-determining step is not the CT reaction but the diffusion of the CT reactants. It is to be noted that the CT rate will decrease with the reaction proceeding according to a simple theory for the CT reaction (Supplementary Note 2). As depicted by the black arrow in Fig. 2d, the diffusion of O_2_ through WS_2_–silica interface is initiated from the edges and substantially hindered because of the narrow interfacial gap. The typical propagation rate for 1LW_silica_ was ~1 μm min^−1^ with a notable variation among samples (Supplementary Fig. 3). On the other hand, the substantially reduced O_2_ sensitivity of 1LW_BN_ (Figs. 1d and 2c) can be attributed to the less hydrophilic nature of hBN that does not hold sufficient water molecules required for the redox reaction (Fig. 2f). In addition, the effective interstitial gap in 1LW_BN_ will be much smaller than that in 1LW_silica_ because of the flatter substrates and consequently enhanced adhesion for the former, and thus interfacial diffusion of oxygen required for CT will be greatly attenuated. This model is also supported by the substantially enhanced O_2_ sensitivity and nondirectional PL enhancement of 1LW_silica_ immersed in water. As shown in Fig. 2b, its PL intensity was increased by ~200% in the presence of dissolved O_2_, but the enhancement did not show any noticeable spatial propagation (Supplementary Movies 3 and 4). Unlike the gas-phase reaction (Fig. 2d), the top surface of WS_2_ is in direct contact with water that serves as a hydrating solvent and thus works as a major CT route as illustrated by the thick blue arrow in Fig. 2e.

### Boosting and quenching of redox reactions

**Fig. 3:**
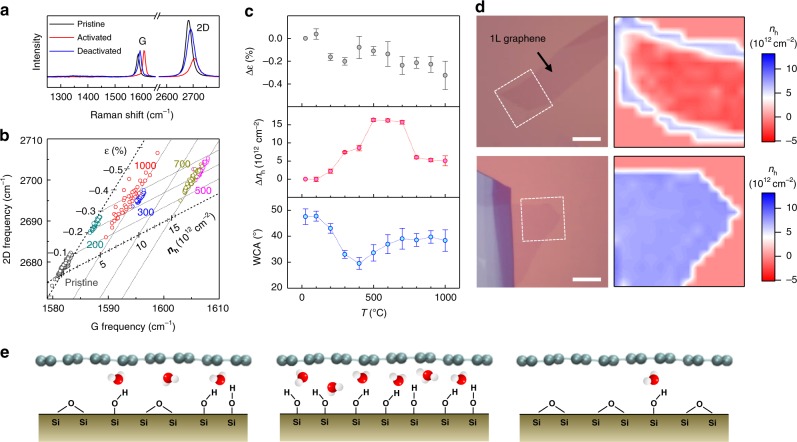
Amplification and suppression of redox reactions. **a** Raman spectra of pristine (black), thermally activated (red; annealed at 500 °C), and deactivated (blue; annealed at 1000 °C) 1L graphene (1LG_silica_). **b** Correlation between G and 2D frequencies of 1LG_silica_ samples annealed at various temperatures (in °C; given next to data). Lattice strain (**ε**) and electrical hole density (***n***_h_) were determined according to ref. ^22^. **c** Changes in lattice strain (top) and hole-density (middle) of graphene; water-contact angle (bottom) of SiO_2_ substrates as a function of annealing temperature (T). Error bars denote standard deviation. **d** Optical micrographs (left) and charge-density images (right) of 1LG_silica_ samples prepared on diethyl zinc-treated (top) and non-treated (bottom) substrates. Both samples were activated by annealing at 400 °C. Scale bars: 10 μm. **e** Schematic representation of air-equilibrated graphene–SiO_2_ interface: pristine (left), hydroxylated by thermal activation (middle), and dehydroxylated by thermal deactivation (right). Equilibrium coverage of interfacial water was determined by effective hydrophilicity of SiO_2_ substrates

To unveil the pivotal role of hydrophilic SiO_2_ surface in the redox reaction, we exploited charge-density-dependent phonon hardening of graphene. As shown in Fig. 3a, G and 2D peaks (*ω*_G_, *ω*_2D_) of 1L graphene/SiO_2_/Si (1LG_silica_) were greatly upshifted and recovered by thermal annealing at 500 and 1000 °C, respectively. Based on the established Raman metrology^22^ of hole density (***n***_h_) and lattice strain (***ε***) shown in Fig. 3b, *ω*_G_ and *ω*_2D_ of samples that were treated at various temperatures were translated into ***n***_h_ and ***ε*** with high accuracy of ~1 × 10^12^ cm^−2^ and ~0.2%, respectively (Fig. 3c). Δ***n***_h_ reached a maximum of 1.0–1.5 × 10^13^ cm^−2^ with slight changes in strain when vacuum-annealed at 400–700 °C (Fig. 3c). Despite many studies^23–27^, however, the mechanistic origin of the thermally activated CT or hole doping has been unclear. In the following section, we propose and show that it is the same redox reaction as Eq. (1), and thermal activation renders SiO_2_ surface hydrophilic enough to bind a certain amount of water molecules. As illustrated in Fig. 3e (left), the surface of silica is terminated with hydrophobic siloxane (Si–O–Si) and hydrophilic silanols (Si–OH). At elevated temperature, the latter transforms into the former without the presence of water (dehydroxylation) and vice versa with water (hydroxylation)^28^. When annealed at <~750 °C, the SiO_2_ surface becomes hydroxylated at the expense of water trapped during sample preparation. When placed in the ambient air, more water molecules are attracted at the graphene–SiO_2_ interface as depicted in Fig. 3e (middle), and electrons of graphene are consumed by the interfacial redox reaction leaving graphene highly hole-doped. When treated at >~750 °C, however, dehydroxylation was more dominant as shown in Fig. 3e (right), and Δ***n***_h_ dropped to 1/3 of the maximum (Fig. 3b, c). Competition between hydroxylation and dehydroxylation was verified by measuring water-contact angles (WCA) for bare substrates (see Supplementary Fig. 4 for raw data). As shown in Fig. 3c, Δ***n***_h_ has a clear correlation with WCA and thus hydrophilicity of substrate surfaces. To further corroborate the hypothesis, we prepared samples in a glove box with interfacial water further minimized by pretreating substrates with diethyl zinc vapor that removes even trace amounts of surface water^29^. The charge-density maps obtained after annealing at 400 °C showed that CT is negligible for diethyl zinc-treated samples unlike non-treated samples (Fig. 3d). This control experiment confirmed that interfacial water is required for the thermal hydroxylation, which leads to the activated CT.

### pH-controlled redox reactions

**Fig. 4:**
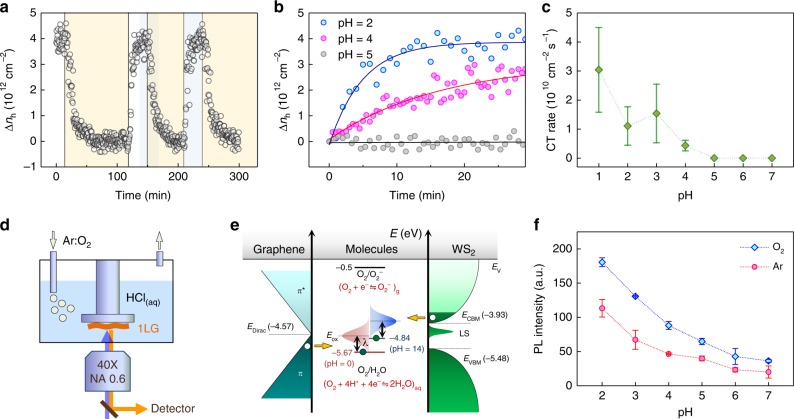
pH-controlled charge transfer. **a** Time-lapse measurements of charge density in 1LG_silica_ in HCl solution (pH = 2) through which Ar (yellow box) and O_2_ (blue box) gases were sparged alternatively. The data near time zero (white box) were obtained with the HCl solution aerated. Δ***n***_h_ was referenced to ***n***_h_ for the Ar flow. **b** pH-dependent kinetics of O_2_-induced rise in charge density of 1LG_silica_ in HCl solutions: pH = 2 (blue), 4 (magenta), and 5 (gray). The solid lines are exponential fits to the data. **c** Initial charge transfer rate per unit area of 1LG_silica_ in HCl solutions of varying pH. **d** Scheme of the optical liquid cell with a gas sparger combined to a micro-spectroscopy setup. **e** Energy-level diagram for redox-governed charge transfer from graphene and WS_2_ to O_2_/H_2_O redox couples. Electron-accepting oxidized states are represented by Gaussian distributions displaced from *E*_F,redox_ by a solvent reorganization energy (*λ*). The Fermi levels of electron donors are at *E*_Dirac_ for graphene and near the conduction band minimum (CBM) for WS_2_. LS represents localized mid-gap states originating from defects. **f** Total PL intensity of 1LW_silica_ modulated by O_2_ dissolved in HCl solution of various pH. Error bars denote standard deviation

We now show that the interfacial CT reaction can be generalized to a pH-dependent redox between 2D materials and O_2_ dissolved in liquid water. High-purity O_2_ or Ar gas was sparged through an optical liquid cell containing HCl solution of a preset pH to control the concentration of dissolved O_2_ (Fig. 4d). When O_2_ was introduced to the Ar-saturated HCl solution of pH = 2, the G and 2D peaks of 1LG_silica_ upshifted (Supplementary Fig. 5), indicating a significant level of hole doping (Δ***n***_h_ ~ 4.0 × 10^12^ cm^−^^2^) (Fig. 4a). Notably, ***n***_h_ decreased reversibly with Ar gas bubbled through the solution, and the doping–undoping cycle could be repeated multiple times. The O_2_-mediated CT was observed at pH = 1–4 but not at pH ≥ 5 (Supplementary Fig. 5). The rise and decay kinetics of ***n***_h_ are highly pH-dependent and self-limited (Fig. 4b, c). A typical initial CT rate is ~1 × 10^10^ cm^−2^s^−1^ at pH = 2 and [O_2_] = 1.3 mM (see Methods). As shown in Fig. 2b, PL signals of WS_2_ also exhibited the same sensitivity toward dissolved O_2_ in water, and the change became more obvious at lower pH (Fig. 4f and Supplementary Fig. 6). Eq. (1) shows that one oxygen molecule may exchange four electrons with other materials in the presence of protons. According to the Gerischer model on CT at an electrode–liquid interface^30^, the direction of CT is determined by energetic alignment between the Fermi level (*E*_F_) of 2D materials and electrochemical potential (*E*_F,redox_) of the redox system as shown in Fig. 4e (Supplementary Note 2). Since *E*_F,graphene_ = −4.57 eV and *E*_F,redox_ = −5.669 + 0.0592pH − 0.0148 log[p(O_2_)], reduction of O_2_ is more favored at lower pH, which leads to increased hole doping of graphene (Supplementary Note 2). On the other hand, the CT is doubly inhibited by a reduction of the reactant and an increase in *E*_F,redox_, when the concentration of O_2_ is decreased. The rate of CT is proportional to the density overlap between occupied states of graphene and empty oxidized states of the redox system, the latter of which is depicted as a Gaussian distribution centered at *E*_ox_ in Fig. 4e. The self-limited CT observed even in the presence of sufficient reactants (Fig. 4b) is due to the CT-induced decrease in *E*_F,graphene_, and can be well described by the Gerischer model (Supplementary Note 2). For WS_2_ that is natively n-doped by various defects including S vacancies^31^, its Fermi level is near the conduction band minimum located at −3.93 eV^32^ that is 0.64 eV higher than that of graphene. Thus, CT from WS_2_ to the redox couples of O_2_/H_2_O is more favorable than from graphene, which is consistent with our finding that the pH threshold for O_2_-induced CT is higher for WS_2_ than that for graphene.

## Discussion

In this work, we have shown that the ambient oxygen reduction reaction is behind the long-standing mystery of the spontaneous and activated CT doping in graphene and WS_2_. Redox couples of O_2_/H_2_O responsible for the CT reside at the interface of 2D materials and substrates, and their 2D diffusion was captured in real time by wide-field PL imaging. The CT reaction can be turned on and off via controlling O_2_ or interfacial water serving as a hydration solvent. The CT rate of 2D materials in contact with liquid water can also be tuned by varying the concentration of dissolved protons or O_2_ as described by the Nernst equation. The presented nanoscopic electrochemistry will pave the way toward efficient control of charge density and the related material properties in 2D, and other low-dimensional materials and devices.

## Methods

### Preparation and treatment of samples

Most of single-layer WS_2_ (1LW_silica_) and graphene (1LG_silica_) samples were prepared by mechanically exfoliating bulk crystals (WS_2_ from 2D semiconductors Inc.; graphite from Covalent Materials Inc. and Naturgraphit GmbH) onto SiO_2_ (285 nm)/Si substrates in the ambient environment. For 1LW_BN_ samples, single layers were first mechanically exfoliated onto polydimethylsiloxane substrates and then dry-transferred onto thin hBN crystals supported on SiO_2_/Si substrates. To minimize a change in spectroscopic signals induced by optical interference, hBN crystals thinner than 3 nm were selected as substrates. To remove interfacial water in 1LG_silica_, we exposed bare substrates briefly to the vapor of diethyl zinc before mechanical exfoliation in a glove box. For thermal activation, samples were annealed at a target temperature for 2 h in a quartz tube furnace that was maintained at a pressure of 3 mTorr.

### Raman and PL measurements

Raman and PL were performed with a homebuilt micro-Raman spectrometer setup^22^. Briefly, monochromatic outputs from solid-state lasers operated at 458 and 514 nm were focused onto samples with a spot size of ∼1 μm by using a microscope objective (40×, numerical aperture = 0.60). Backscattered PL and Raman signals were collected with the same objective and guided to a spectrometer equipped with a liquid nitrogen-cooled CCD detector. Overall spectral accuracy was better than 5 and 1 cm^−1^ for PL and Raman measurements, respectively. To avoid significant photoinduced effects, we maintained the average power of the excitation beam below 6 μW for WS_2_ and 400 μW for graphene samples.

For wide-field PL imaging, the collimated green laser beam was focused at the back-focal plane of the objective with a plano-convex lens (focal length = 400 mm) after three-times expansion with a Galilean beam expander. The average power of the wide-field excitation was maintained below 1.5 mW that was illuminated onto an area with a diameter of ~100 μm. PL signals in the range between 1.9 and 2.1 eV mostly contributed to the PL images recorded with the CCD detector.

### Control of gas and liquid environments

For optical measurements in controlled gas environments, samples were mounted in a custom-made optical gas cell with precise controls over the flow rates of Ar (20–1000 mL min^−1^) and O_2_ (5–250 mL min^−1^). Unless otherwise noted, flow rates for dry gases were 1000 and 250 mL min^−1^ for Ar and O_2_, respectively. Relative humidity (RH) inside the gas cell was monitored by a serially connected hygrometer and could be controlled between 5 and 90% by varying the mixing ratio of an additional Ar gas line that passed through a water bubbler (Supplementary Fig. 1a). The temporal change in RH upon injection of wet gas could be well fitted with two exponential functions with time constants of 15 ± 2 and 116 ± 40 s. The fast component is limited by the finite flow rate through the gas manifold, and the slower one is related to adsorption kinetics of water on the inner walls of the manifold and gas cell. The former time constant set an upper bound for the average arrival time for the dry gas experiments since the gas manifold was smaller for the dry gas case. The equilibrium RH values were in a linear relation with the mixing ratio of the wet Ar gas (Supplementary Fig. 1b), showing good controllability.

For in situ measurements in aqueous solutions, samples were placed in a custom-designed Teflon-based optical liquid cell with a gas sparger. The concentration of dissolved oxygen was controlled by flowing high-purity O_2_ or Ar gases at 250 mL min^−1^ through the sparger, which is an efficient method to saturate or eliminate dissolved oxygen^33^. When saturated under 1 atm O_2_ gas, a simple estimation by using Henry’s law at 298 K predicts that the concentration of O_2_ will reach 1.3 mM. On the other hand, a 5-min or longer sparging of inert gas leads to a residual concentration of ~15 μM or less^33^. pH of aqueous solutions was varied with hydrochloric acid.

### Contact-angle measurements

The hydrophilicity of SiO_2_/Si substrates was quantified by WCA to reveal competition between thermally activated surface hydroxylation and dehydroxylation^28^ in the presence of water vapor. Bare SiO_2_/Si substrates were annealed at various temperatures for 2 h in the tube furnace filled with water vapor and O_2_ gases. Within 20 min after the treatments, WCA of the substrates was measured in the static mode with an optical tensiometer (SmartDrop, Femtobiomed Inc.) in ambient conditions. The volume of water droplets was maintained in the range of 2.5–3 μL.

## Supplementary information

Supplementary Information

Supplementary Movie 1

Supplementary Movie 2

Supplementary Movie 3

Supplementary Movie 4

## Data Availability

The data that support the findings of this study are available in the Supplementary Information and from the corresponding author upon reasonable request.
