# Supplementary material for: Redox-governed charge doping dictated by interfacial diffusion in two-dimensional materials
Source: Nat Commun. 2019 Oct 30;10:4931. doi: 10.1038/s41467-019-12819-w (PMC6821894; doi:10.1038/s41467-019-12819-w)
Supplement: Supplementary file 1 — Supplementary Information [file 41467_2019_12819_MOESM1_ESM.pdf]

Supplementary Information for

Redox-governed charge doping dictated by interfacial diffusion in two-dimensional materials

Kwanghee Park, Haneul Kang, Seonghyun Koo, DaeEung Lee and Sunmin Ryu*

Correspondence to: sunryu@postech.ac.kr

This PDF file includes:

Supplementary Notes
Supplementary Figures (1 to 6)
Supplementary Table 1
Legends for Supplementary Movies (1 to 4)
Supplementary References

Other Supplementary information for this manuscript includes the following:

Supplementary Movies (1 to 4)

Supplementary Notes

1. Estimation of carrier density of 1L WS_2

The density of free charge carriers (e and h for electrons or holes, respectively) in single layer WS_2 was estimated from the spectral weight of excitons (X^0) and trions (X^- or X^+) in their PL spectra. While there have been similar approaches (*1, 2*), a detailed account is given to clarify its derivation and correct numerical errors.

First, the number densities of the free charge carriers and quasi-particles were related to the dissociation energy (E_{diss}) of trions determined from the PL measurements. Under a constant photoirradiation, trions were assumed to be in thermodynamic equilibrium with excitons and free charge carriers.

$$X^- \leftrightarrow X^0 + e \quad (A1); \quad X^+ \leftrightarrow X^0 + h \quad (A2)$$

For the equilibrium of the negative trion (Equation A1), the particle numbers (N_J) of the three species (J: X^-, X^0, e) satisfy the following relation with their partition functions (Z_J) (*3*).

$$\frac{N_{X^0}N_e}{N_{X^-}} = \frac{Z_{X^0}Z_e}{Z_{X^-}} = \frac{z_{X^0}^T z_e^T}{z_{X^-}^T}\frac{z_{X^0}^E z_e^E}{z_{X^-}^E} \quad \text{(A3)}$$

, where Z_J is given as a product of translational (z_J^T) and electronic (z_J^E) components, since there is no vibrational and rotational contribution for the three species. For the 2-dimensional system, $z_J^T = \frac{A}{\Lambda_J{}^2}$, where A and Λ_J are the area of the system and the thermal wavelength of J, respectively. When only the ground state contribution is considered for the electronic part and the zero of electronic energy is referenced to a completely separated pair of an electron at the Fermi level and exciton at rest in its ground state, the energy of trion is $-E_{diss}$ or minus of dissociation energy of trion. Then, the product of z_J^E in Equation A3 is given as follows (*3*).

$$\frac{z_{X^0}^E z_e^E}{z_{X^-}^E} = \frac{g_{X^0}g_e}{g_{X^-}}\exp\left(-\frac{E_{diss}}{k_BT}\right) = \frac{4\cdot 2}{2}\exp\left(-\frac{E_b}{k_BT}\right) = 4\exp\left(-\frac{E_{diss}}{k_BT}\right) \quad \text{(A4)}$$

, where g_J is degeneracy associated with spin multiplicity (*4*).

Now the equilibrium constant (K) of Equation A1 defined under the law of mass action can be expanded as follows (*5*).

$$K = \frac{n_{X^0}n_e}{n_{X^-}} = \frac{1}{A}\frac{N_{X^0}N_e}{N_{X^-}} = 4\frac{\Lambda_{X^-}{}^2}{\Lambda_{X^0}{}^2\Lambda_e{}^2}\exp(-\frac{E_{diss}}{k_BT}) \quad \text{(A5)}$$

, where n_J is the 2D number density of each species. Since Λ_J is $\sqrt{(h^2/2\pi m_J k_B T)}$, where m_J is effective mass, the following Equation A6 can be obtained.

$$K = \frac{n_{X^0} n_e}{n_{X^-}} = \frac{2k_B T m_{X^0} m_e}{\pi \hbar^2 m_{X^-}} \exp\left(-\frac{E_{diss}}{k_B T}\right) \quad (A6)$$

As a second step to determine n_e, the number density ratio between the two quasi-particles in Equation A6 was replaced by their PL intensity ratio based on a simple three-level model (*6*). A set of rate equations for the density of exciton and trion can be expressed as follows.

$$\frac{dn_{X^0}}{dt} = G - (\Gamma_{X^0} + k_{X^-}) n_{X^0} \quad (A7)$$

$$\frac{dn_{X^-}}{dt} = k_{X^-} n_{X^0} - \Gamma_{X^-} n_{X^-} \quad (A8)$$

, where G is the optical generation rate of exciton, k_{X^-} is the formation rate of trion from exciton, and Γ_{X^0} and Γ_{X^-} are the decay rates of exciton and trion, respectively. Under the steady-state approximation, the following solutions for n_J can be obtained.

$$n_{X^0} = \frac{G}{\Gamma_{X^0} + k_{X^-}} \quad (A9); \qquad n_{X^-} = \frac{k_{X^-}}{\Gamma_{X^-}} \frac{G}{\Gamma_{X^0} + k_{X^-}} \quad (A10)$$

The observed PL intensities of the exciton (I_{X^0}) and trion (I_{X^-}) are linearly related to their densities as follows.

$$I_{X^0} = A\gamma_{X^0} n_{X^0} = \frac{AG\gamma_{X^0}}{\Gamma_{X^0} + k_{X^-}} \quad (A11)$$

$$I_{X^-} = A\gamma_{X^-} n_{X^-} = \frac{k_{X^-}}{\Gamma_{X^-}} \frac{AG\gamma_{X^-}}{\Gamma_{X^0} + k_{X^-}} \quad (A12)$$

, where A is a proportionality constant, and γ_{X^0} and γ_{X^-} are the radiative decay rates of exciton and trion, respectively. Then, the intensity ratio of trion to exciton, I_{X^-}/I_{X^0}, can be related to Equation A6 using Equations A11 & A12 as follows.

$$\frac{I_{X^-}}{I_{X^0}} = \frac{\gamma_{X^-} n_{X^-}}{\gamma_{X^0} n_{X^0}} = \frac{\gamma_{X^-}}{\gamma_{X^0}} \frac{\pi \hbar^2 n_e m_{X^-}}{2k_B T m_{X^0} m_e} \exp\left(\frac{E_{diss}}{k_B T}\right) \quad (A13)$$

When rearranged, Equation 13 reveals n_e in terms of experimental observables as follows.

$$n_e = \frac{2k_B T m_{X^0} m_e}{\pi \hbar^2 m_{X^-}} \exp\left(-\frac{E_{diss}}{k_B T}\right) \left(\frac{\gamma_{X^-}}{\gamma_{X^0}}\right)^{-1} \frac{I_{X^-}}{I_{X^0}} \quad (A14)$$

In order to evaluate n_e using Equation A14, the ratio for effective masses ($m_{X^0} m_e / m_{X^-}$) was taken to be 0.24m_0, an average for two theoretical predictions: m_e = 0.44m_0, m_h = 0.45m_0 (*7*); m_e = 0.27m_0, m_h = 0.32m_0, where m_0 is the rest mass of an electron (*8*). To our knowledge, radiative decay rates for both quasi-particles have not been measured directly. Ross et al. suggested that

both quantities are equal based on their steady-state PL measurements (*2*). Accordingly assuming the ratio of radiative decay rates for both quasi-particles ($\gamma_{X^-}/\gamma_{X^0}$) is unity, Equation A14 leads to the following equation for n_e (in cm^{-2}).

$$n_e = 5.2 \times 10^{12} \frac{I_{X^-}}{I_{X^0}} \exp\left(-\frac{E_{diss}}{k_B T}\right) \quad (A15)$$

We note that there have been indirect estimations of the ratio using chemical doping based on a Langmuir isotherm model. Adopting $\gamma_{X^-}/\gamma_{X^0}$ = 0.14, an average value of three (0.15, 0.20 and 0.06) that are available in the literature (*6, 9, 10*), n_e (in cm^{-2}) is given as follows and will be ~7 times larger than what Equation A15 predicts.

$$n_e = 3.7 \times 10^{13} \frac{I_{X^-}}{I_{X^0}} \exp\left(-\frac{E_{diss}}{k_B T}\right) \quad (A16)$$

As summarized in Supplementary Table 1, our spectroscopic data and a previous study using electrically doped systems are more consistent with Equation A15 than Equation A16. As shown in Fig. 1e, I_{X^-}/I_{X^0} (E_{diss}) decreased from 3.6 (37 meV) in Ar to 0.5 (27 meV) in wet Ar:O_2. The difference in n_e estimated from Equation A16 (2.4x10^{13} cm^{-2}) is ~7 times larger than that from Equation A15 (3.7x10^{12} cm^{-2}). In order to judge between the two expressions for n_e, two independent estimations were made. First, Δn_e was determined from ΔE_{diss}, since E_{diss} is directly proportional to E_F (*11*). Δn_e = -2.9x10^{12} cm^{-2} was obtained from 10 meV decrease in E_{diss} using Equation A17 (*11*) where m_e = 0.35m_0 (*7, 8*).

$$E_F = \frac{\hbar^2 \pi n_e}{2 m_e} \quad (A17)$$

Secondly, a similar value was obtained from the PL data modulated by electrically gating 1L WS_2 in a field-effect transistor geometry (thickness of SiO_2 gate dielectric = 300 nm, gate capacitance = 115 aF·µm^{-2}) (*12*). To vary I_{X^-}/I_{X^0} from 3.6 to 0.5, the back gate voltage was lowered by ~45 V, which corresponds to Δn_e = -3.2x10^{12} cm^{-2}. These two results are consistent within 20% with Equation A15, but deviate significantly from Equation A16. However, one needs to be cautious in using Equation A15 because the radiative decay rates of both quasi-particles are dependent on charge density (*13*). Because γ_{X^0} (γ_{X^-}) decreases (increases) with increasing charge density, n_e determined from Equation A15 will be overestimated (underestimated) at high (low) charge density.

In summary, the electron density of 1L WS_2 supported on SiO_2 decreases by ~3x10^{12} cm^{-2} during the ambient redox reaction (Fig. 1e). In addition, comparison with other data (Supplementary Table 1) indicated that the radiative decay rates of X^0 and X^- are almost equivalent. However, our data in Fig. 1e and Supplementary Table 1 would be better described by $\gamma_{X^-}/\gamma_{X^0}$ = 1.3, assuming the validity of the three-level model obeying the law of mass action.

Supplementary Table 1. Estimation of charge density of 1L WS_2. The optical data were obtained from Fig. 1e.

atmospheric conditions	I_{X^-}/I_{X^0} [Fig. 1e]	E_{diss} (meV) [Fig. 1e]	n_e (cm^{-2}) [Eqn. A15]	n_e (cm^{-2}) [Eqn. A16]	electrical measurements [Ref. 12]
Ar	~3.6	~37	~4.7x10^{12}	~3.3x10^{13}	-
wet Ar:O_2	~0.5	~27	~1.0x10^{12}	~7.1x10^{12}	-
Δn_e (cm^{-2})	-	-2.9x10^{12}	-3.7x10^{12}	-2.4x10^{13}	-3.2x10^{12}

2. Nernst equation for interfacial CT based on Gerischer Model

The charge transfer observed in the current work occurs between graphene or WS_2 and the molecular reactants O_2/H_2O redox couples, which undergo the following reaction: $O_2 + 4H^+ + 4e^- \leftrightarrow 2H_2O$ (under acidic conditions). Such an electrochemical charge doping, also named as surface transfer doping, dates back to the late 1980s when extremely high surface conduction was observed for hydrogenated diamond surfaces (*14, 15*). F. Maier et al. proposed its electrochemical nature (*16*), and V. Chakrapani et al. (*17*) validated that the very redox reaction was responsible for the charge doping in the hydrogen-passivated diamonds. Its mechanistic validation also led to revelation of similar phenomena in other material systems including GaN, ZnO, and carbon nanotubes (*18, 19*). The idea was further refined by P. Levesque et al. (*20*) by using the Gerischer model (*21, 22*) that was developed for charge transfer reaction occurring at electrodes in solutions. Below, we set up a Nernst equation for interfacial charge transfer also adopting the above approach to explain charge transfer in WS_2 and graphene systems.

2.1. Rate of charge transfer. Assume a general redox half-cell reaction:

$$\boldsymbol{ox} + ze^- \leftrightarrow \boldsymbol{red} \quad (B1)$$

, where ***ox*** and ***red*** represent solvated oxidized and reduced molecular species, respectively, and z is the number of electrons. Electron transfer occurs from an occupied energy state of the solid electrode to an unoccupied state (***ox***) of the molecular redox system. Since the transfer is faster than the rearrangement of the solvent molecules, the transfer rate is determined by the degree of overlap between densities of energy states on both sides as follows (also schematically shown in Fig. 4e).

$$transfer\ rate \ \propto \ c_{ox} \int_{-\infty}^{\infty} f(E)\rho(E)W_{ox}(E)dE \quad (B2)$$

, where c_{ox} is the concentration of ***ox*** species, $f(E)$ and $\rho(E)$ are the distribution functions of the Fermi-Dirac and energy states in the electrode, respectively, and $W_{ox}(E)$ is the distribution function of solvated ***ox*** species in the redox system. $W_{ox}(E)$ is given as a Gaussian function (*21*) as follows

$$W_{ox}(E) = W_0 \exp\left\{\frac{-(E - E_{ox})^2}{4k_B T\lambda}\right\} \quad (B3)$$

, where W_0 is a normalization factor, E_{ox} is energy level of ***ox*** species, k_B is Boltzmann constant, T is temperature and λ is reorganization energy. $E_{ox(red)}$ is higher (lower) than the Fermi level of the redox system $E_{F,redox}$ (or electrochemical potential of electrons in the redox system) by λ, which was set to be 1 eV for the aqueous environment (*21*).

2.2. Energetics of electron donor & acceptor. In order to relate the solid electrodes with molecular species, all relevant energy levels were referenced to the vacuum level (E_v) as shown in Fig. 4e. The Fermi level of graphene is -4.57 eV (*23*), and edge energies of the valence and conduction bands of WS_2 are -5.48 and -3.93 eV, respectively (*24*). The mid-gap localized state (LS) originating from S-vacancy and inducing n-type doping in WS_2 is located ~0.5 eV below the conduction band minimum (*25*).

The energy levels of O_2/H_2O redox system can be determined by the Nernst equation. The reduction potential (E_{hc}) of the half-cell reaction (Equation B1) is given as

$$E_{hc} = E_{hc}^{o} - \frac{RT}{zF}\ln\frac{c_{red}}{c_{ox}} \qquad (B4)$$

, where E_{hc}^{o} is the standard reduction potential (1.229 V) for Equation 1 in the main text, R is the gas constant, F is the Faraday constant, and c_{ox} and c_{red} are the concentrations of reactants (***ox***) and products (***red***), respectively. Then, E_{hc} can be related to $E_{F,redox}$ that is referenced to the vacuum level as follows:

$$E_{F,redox} = \mu_{e(SHE)} - \mathrm{e}\cdot E_{hc} = \mu_{e(SHE)} - \mathrm{e}\cdot E_{hc}^{o} + \frac{k_B T}{z}\ln\frac{c_{red}}{c_{ox}} \qquad (B5)$$

, where $\mu_{e(SHE)}$ (-4.44 eV) is the potential energy of the standard hydrogen electrode (SHE) with respect to the vacuum level. The equation for $E_{F,redox}$ of the O_2/H_2O redox system given below indicates that lower pH and higher pressure of O_2, $\mathrm{p(O_2)}$, lead to lower $E_{F,redox}$ and thus more favorable electron transfer.

$$E_{F,redox} = -5.669 + \frac{0.0592}{4}[4\mathrm{pH} - \log \mathrm{p(O_2)}] \quad (B6)$$

2.3. Kinetics of charge transfer. The Equation B2 indicates that the transfer rate decreases as electron transfer proceeds and the Fermi level (E_F) of the electron donor (graphene and WS_2) is subsequently lowered. For graphene, E_F can be evaluated from hole density (n_h) as follows (*26*):

$$E_F = -4.57 - \hbar|v_F|\sqrt{\pi n_h} \quad (B7)$$

This reasoning leads to the notion of effective threshold energy for acceptors (E_{th}). In principle, all electrons of the donor electrode below E_F may have a non-zero probability of transfer in Equation B2. However, electrons near E_F contribute mostly because of the rapidly varying Gaussian energy distribution of the acceptor (W_{ox}). As E_F of the donor decreases far below E_{ox}, the transfer rate in Equation B2 is too small to be detected, where E_F can be set as E_{th}. Accordingly, E_{th} can be assumed to obey the same dependence on pH and $\mathrm{p(O_2)}$ as $E_{F,redox}$ in Equation B6.

$E_{F,redox}$ for 1 atm O_2 gas increases from -5.669 eV at pH = 0 to -4.840 eV at pH =14 with a rate of 59.2 meV/pH. Since the energy distribution of the acceptor (W_{ox}) is centered at E_{ox} (1 eV above $E_{F,redox}$), its overlap with the Fermi level of charge-neutral graphene and thus E_{th} vary appreciably depending on pH. In Fig. 4b, Δn_h for pH = 2 reached an asymptotic value of ~4x10^{12} cm^{-2}. Since E_F of -4.83 eV corresponds to the charge density according to Equation B7, it can be equated to E_{th} at pH = 2. As pH was increased in Fig. 4b, the transfer rate decreased since E_{th} increases at a rate of 59.2 meV/pH. Since E_F of WS_2 with native n-type dopants is near the conduction band minimum (-3.93 eV), electron transfer to the donor is allowed at much higher pH as shown in Fig. 4f. Since E_{th} is estimated to be -4.09 at pH = 14, O_2-induced PL enhancement in WS_2 is expected to occur at pH > 7 (Fig. 4f).

It is also to be noted that $p(O_2)$ determining the concentration of dissolved O_2, [O_2], plays a double role. First, the transfer rate is directly proportional to [O_2] (Equation B2). Second, $E_{F,redox}$ and thus E_{th} (transfer rate) will be lowered (enhanced) with increasing $p(O_2)$ (Equation B6).

Supplementary Figures

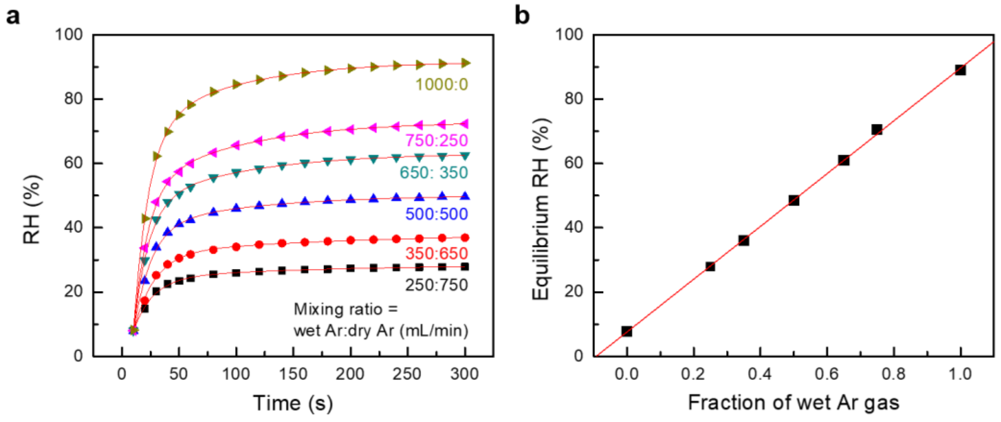

Supplementary Figure 1. Control of relative humidity. (a) Temporal change in relative humidity (RH) after injection of wet Ar gas into the optical gas cell pre-equilibrated with dry Ar gas for various mixing ratios. The data were well fitted with double exponential functions (solid lines), where the short and long time constants were 14.8 ± 2.3 s and 116 ± 40 s. (b) Equilibrium RH values as a function of the fraction of wet Ar. The solid line is a linear fit to the data.

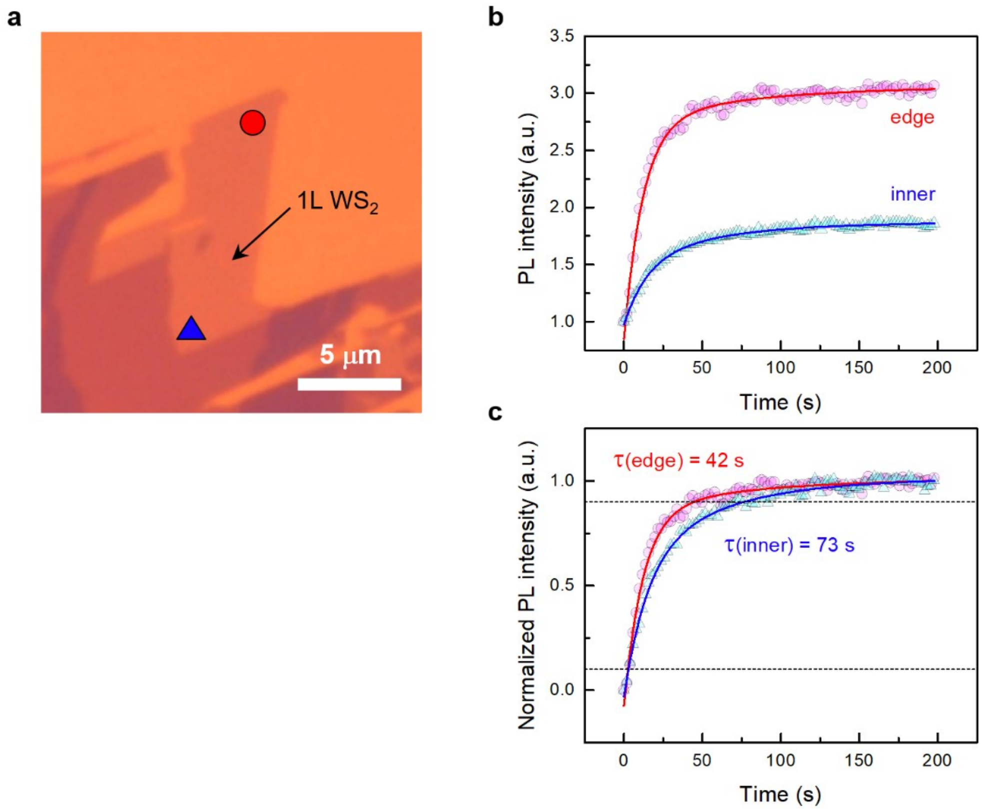

Supplementary Figure 2. Site-dependent kinetics of charge transfer. (a) Optical micrograph of 1L WS_2 on SiO_2/Si substrate. Time-lapse PL measurements were made at an edge (circle) and an inner (triangle) spots to examine site dependence of effects of gases. (b) Total PL intensity (I_t) obtained for both spots after Ar:O_2 (4:1) gas replaced Ar gas at time zero. The signal was normalized with that for time zero. The solid lines are double-exponential fits to the data. (c) The data in (b) shown after rescaling in intensity for clear comparison. An effective rising time (τ), defined over 10 ~ 90% intervals (dashed lines), was much smaller for edges than inner spots. A significant fraction of τ is due to the finite response time during the gas exchange (see Methods).

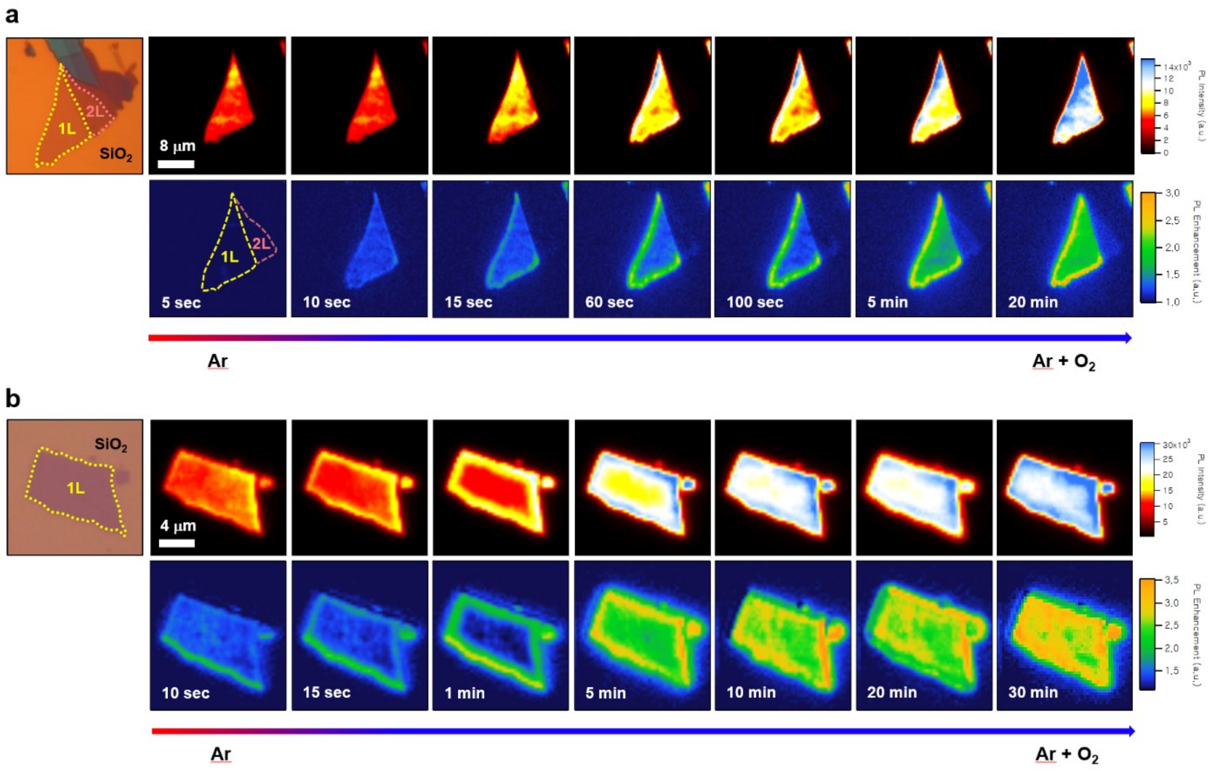

Supplementary Figure 3. Time-lapse photoluminescence images of two additional 1L WS_2 samples. Upper rows of (a & b) present optical micrographs and time-stamped wide-field PL images obtained after Ar:O_2 (4:1) gas replaced Ar gas flow at time zero. PL enhancement images given in the bottom rows were obtained by dividing PL images with that for time zero. The enhancement images in (a) showed that the 2L area also exhibited edge-to-center propagation of enhancement.

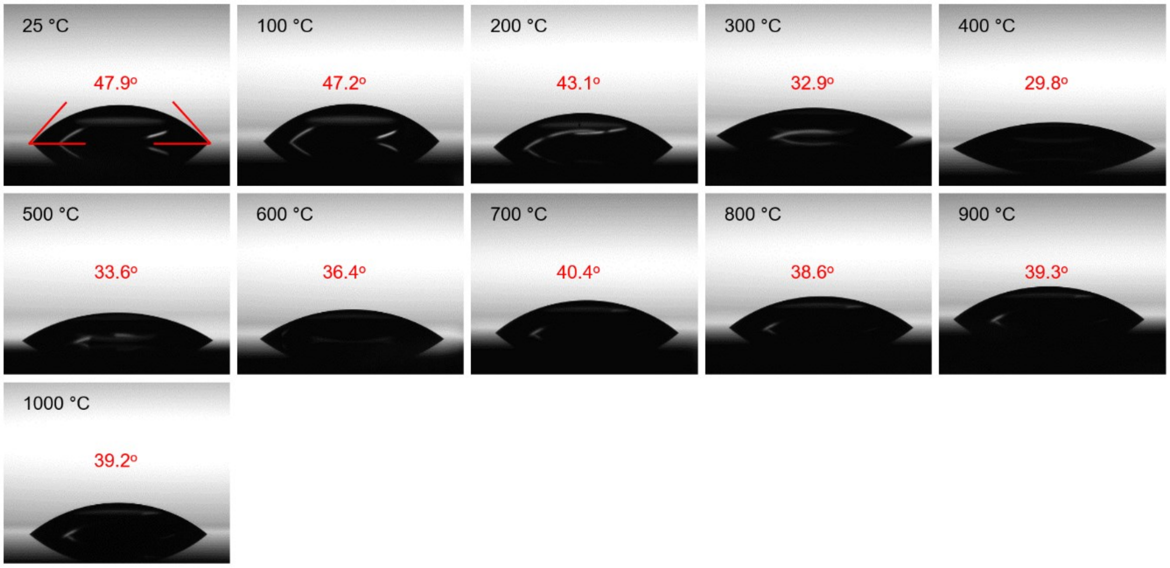

Supplementary Figure 4. Water contact angles of thermally activated SiO_2 substrates. Optical images of water drops on substrates were obtained and analyzed with a tensiometer (see Methods for details). Annealing temperatures and contact angles are given in each image.

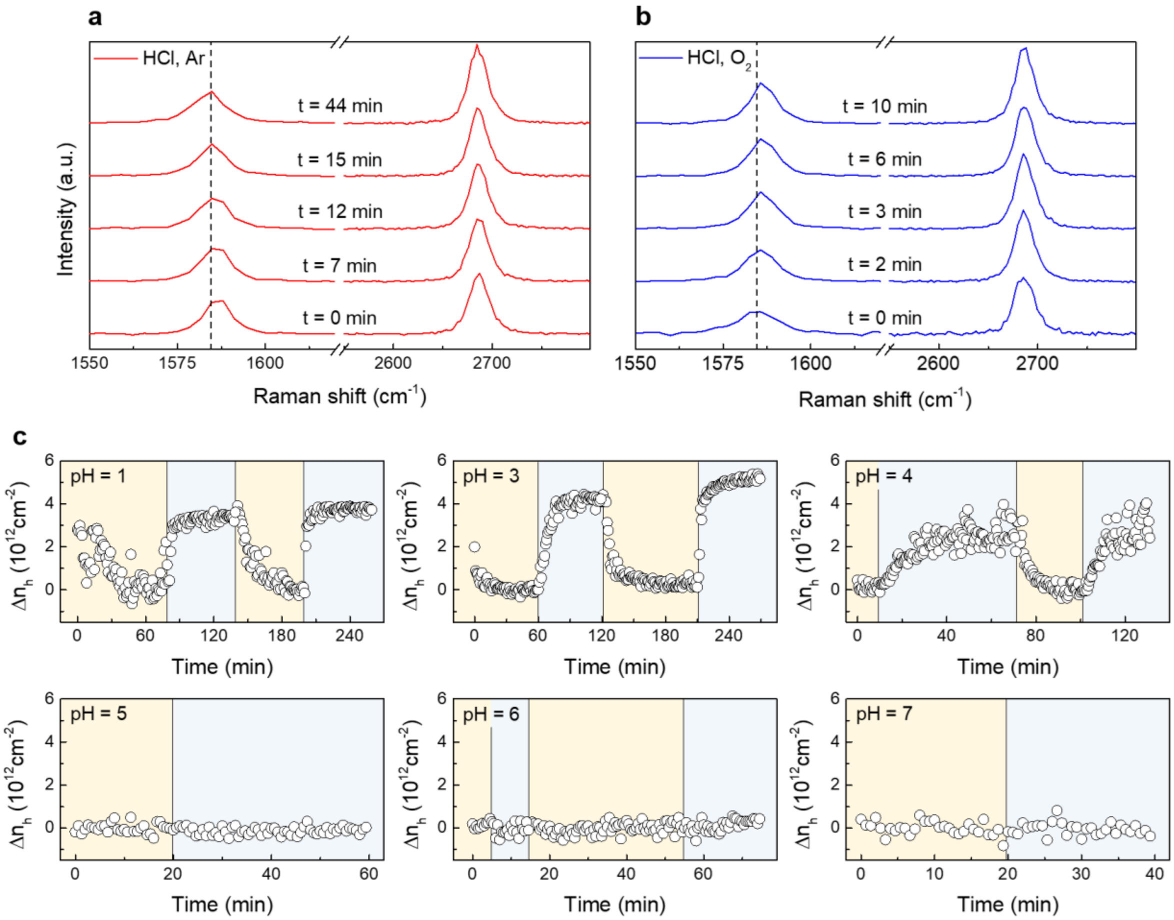

Supplementary Figure 5. Effects of dissolved O_2 on charge density of graphene in HCl solution. (a) Time-lapse Raman spectra (from bottom to top) of pristine 1L graphene/SiO_2/Si obtained as Ar gas was sparged through O_2-saturated HCl solution of pH = 2 at time zero. (b) Time-lapse Raman spectra (from bottom to top) obtained as O_2 gas was sparged through Ar-saturated HCl solution of pH = 2 at time zero. (c) Change in hole density ($\boldsymbol{n}_h$) of graphene immersed in HCl solution of various pH in response to sparging Ar (yellow box) or O_2 (blue box) alternatively.

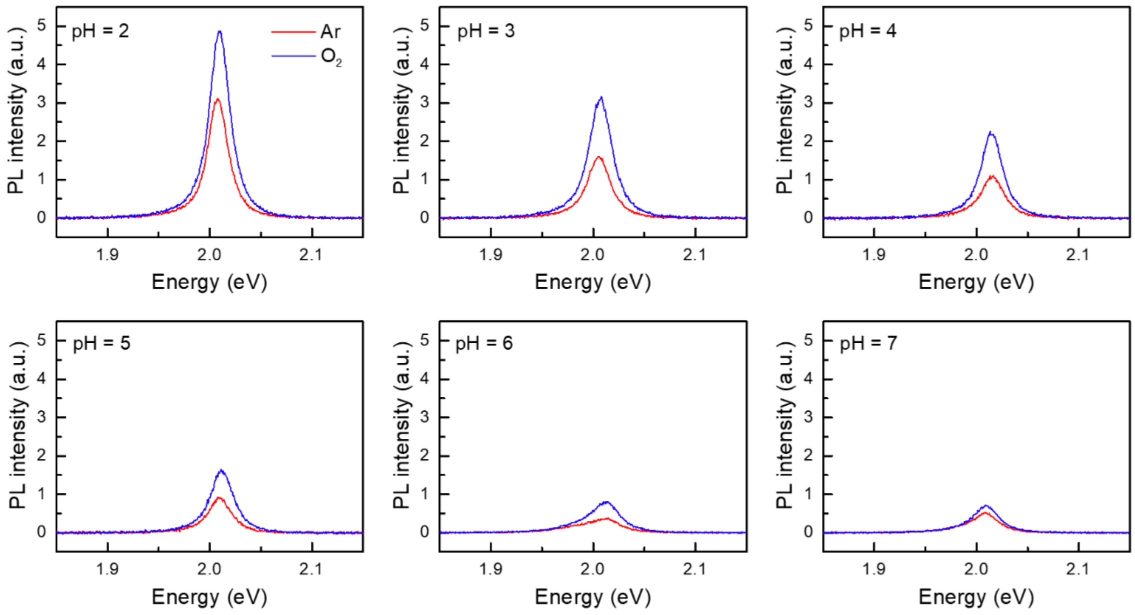

Supplementary Figure 6. Effects of dissolved O_2 on charge density of WS_2 in HCl solution. PL spectra of 1L $WS_2/SiO_2/Si$ obtained in HCl solution of various pH after sparging Ar (red) or O_2 (blue) for 60 min.

Legends for Supplementary Movies

Movie 1. Time-lapse PL enhancement image of WS_2 in O_2 gas. 1L $WS_2/SiO_2/Si$ was pre-equilibrated in Ar gas until replacement with Ar:O_2 (4:1) gas at time zero. The timestamp in each frame, *aa*:*bb*:*cc*, stands for *aa* minutes *bb* seconds *cc* milliseconds. Some snapshots of the movie and the definition of PL enhancement are given in Fig. 2a.

Movie 2. Time-lapse PL enhancement image of WS_2 in Ar gas. 1L $WS_2/SiO_2/Si$ was pre-equilibrated in O_2 gas until replacement with Ar gas at time zero.

Movie 3. Time-lapse PL enhancement image of WS_2 in water sparged with O_2. 1L $WS_2/SiO_2/Si$ immersed in deionized water was pre-sparged with Ar gas until replacement with O_2 gas at time zero. Some snapshots of the movie are given in Fig. 2b.

Movie 4. Time-lapse PL enhancement image of WS_2 in water sparged with Ar. 1L $WS_2/SiO_2/Si$ immersed in deionized water was pre-sparged with Ar gas until the introduction of O_2 gas at time zero.

Supplementary References

1. J. Siviniant, D. Scalbert, A. V. Kavokin, D. Coquillat, J. P. Lascaray, Chemical equilibrium between excitons, electrons, and negatively charged excitons in semiconductor quantum wells. *Phys. Rev. B* **59**, 1602-1604 (1999).
2. J. S. Ross *et al.*, Electrical control of neutral and charged excitons in a monolayer semiconductor. *Nat. Commun.* **4**, 1474 (2013).
3. D. A. McQuarrie, J. D. Simon, *Physical Chemistry: A Molecular Approach*. (University Science Books;, 1997).
4. A. Ron *et al.*, Thermodynamics of free trions in mixed type GaAsAlAs quantum wells. *Solid State Commun.* **97**, 741-745 (1996).
5. A. Esser, E. Runge, R. Zimmermann, W. Langbein, Trions in GaAs Quantum Wells: Photoluminescence Lineshape Analysis. *Phys. Status Solidi A* **178**, 489-494 (2000).
6. S. Mouri, Y. Miyauchi, K. Matsuda, Tunable Photoluminescence of Monolayer MoS2 via Chemical Doping. *Nano Lett.* **13**, 5944-5948 (2013).
7. A. Ramasubramaniam, Large excitonic effects in monolayers of molybdenum and tungsten dichalcogenides. *Phys. Rev. B* **86**, 115409 (2012).
8. H. Shi, H. Pan, Y.-W. Zhang, B. I. Yakobson, Quasiparticle band structures and optical properties of strained monolayer MoS2 and WS2. *Phys. Rev. B* **87**, 155304 (2013).
9. N. Peimyoo *et al.*, Chemically Driven Tunable Light Emission of Charged and Neutral Excitons in Monolayer WS2. *ACS Nano* **8**, 11320-11329 (2014).
10. Y. Tao *et al.*, Bright monolayer tungsten disulfide via exciton and trion chemical modulations. *Nanoscale* **10**, 6294-6299 (2018).
11. K. F. Mak *et al.*, Tightly bound trions in monolayer MoS2. *Nat. Mater.* **12**, 207-211 (2013).
12. J. Shang *et al.*, Observation of Excitonic Fine Structure in a 2D Transition-Metal Dichalcogenide Semiconductor. *ACS Nano* **9**, 647-655 (2015).
13. S. Tongay *et al.*, Broad-Range Modulation of Light Emission in Two-Dimensional Semiconductors by Molecular Physisorption Gating. *Nano Lett.* **13**, 2831-2836 (2013).
14. M. I. Landstrass, K. V. Ravi, Hydrogen passivation of electrically active defects in diamond. *Appl. Phys. Lett.* **55**, 1391-1393 (1989).
15. S. A. Grot *et al.*, The effect of surface treatment on the electrical properties of metal contacts to boron-doped homoepitaxial diamond film. *IEEE Electron Device Lett.* **11**, 100-102 (1990).
16. F. Maier, M. Riedel, B. Mantel, J. Ristein, L. Ley, Origin of Surface Conductivity in Diamond. *Phys. Rev. Lett.* **85**, 3472-3475 (2000).
17. V. Chakrapani *et al.*, Charge transfer equilibria between diamond and an aqueous oxygen electrochemical redox couple. *Science* **318**, 1424-1430 (2007).
18. V. Chakrapani *et al.*, Electrochemical Pinning of the Fermi Level: Mediation of Photoluminescence from Gallium Nitride and Zinc Oxide. *J. Am. Chem. Soc.* **130**, 12944-12952 (2008).
19. V. Chakrapani, G. U. Sumanasekera, B. Abeyweera, A. Sherehiy, J. C. Angus, Electrochemically Induced p-Type Conductivity in Carbon Nanotubes. *Ecs Solid State Letters* **2**, M57-M60 (2013).
20. P. L. Levesque *et al.*, Probing Charge Transfer at Surfaces Using Graphene Transistors. *Nano Lett.* **11**, 132-137 (2011).
21. R. Memming, *Semiconductor electrochemistry*. (Wiley-VCH Verlag, 2001).

22. A. J. Bard, L. R. Faulkner, *Electrochemical methods: Fundamentals and applications*. (John Wiley & Sons, ed. 2nd edition, 2001).
23. Y. J. Yu *et al.*, Tuning the Graphene Work Function by Electric Field Effect. *Nano Lett.* **9**, 3430-3434 (2009).
24. J. Kang, S. Tongay, J. Zhou, J. Li, J. Wu, Band offsets and heterostructures of two-dimensional semiconductors. *Appl. Phys. Lett.* **102**, 012111 (2013).
25. S. Salehi, A. Saffarzadeh, Atomic defect states in monolayers of MoS2 and WS2. *Surf. Sci.* **651**, 215-221 (2016).
26. Y. Zhang, Y.-W. Tan, H. L. Stormer, P. Kim, Experimental observation of the quantum Hall effect and Berry's phase in graphene. *Nature* **438**, 201-204 (2005).
